# Case Report: Histopathology and Prion Protein Molecular Properties in Inherited Prion Disease With a *De Novo* Seven-Octapeptide Repeat Insertion

**DOI:** 10.3389/fncel.2020.00150

**Published:** 2020-07-08

**Authors:** Ignazio Cali, Laura Cracco, Dario Saracino, Rossana Occhipinti, Cinzia Coppola, Brian Stephen Appleby, Gianfranco Puoti

**Affiliations:** ^1^Department of Pathology, School of Medicine, Case Western Reserve University, Cleveland, OH, United States; ^2^National Prion Disease Pathology Surveillance Center (NPDPSC), School of Medicine, Case Western Reserve University, Cleveland, OH, United States; ^3^Department of Pathology and Laboratory Medicine, School of Medicine, Indiana University, Indianapolis, IN, United States; ^4^Division of Neurology, University of Campania “Luigi Vanvitelli”, Caserta, Italy; ^5^Prion Disease Diagnosis and Surveillance Center (PDDSC), University of Campania “Luigi Vanvitelli”, Caserta, Italy; ^6^Department of Physiology and Biophysics, School of Medicine, Case Western Reserve University, Cleveland, OH, United States; ^7^Department of Neurology, School of Medicine, Case Western Reserve University, Cleveland, OH, United States; ^8^Department of Psychiatry, School of Medicine, Case Western Reserve University, Cleveland, OH, United States

**Keywords:** genetic, histopathology, Prion protein (PrP), insertion mutation, conformation

## Abstract

The insertion of additional 168 base pair containing seven octapeptide repeats in the prion protein (PrP) gene region spanning residues 51–91 is associated with inherited prion disease. In 2008, we reported the clinical features of a novel *de novo* seven-octapeptide repeat insertion (7-OPRI) mutation coupled with codon 129 methionine (M) homozygosity in the PrP gene of a 19-year-old man presenting with psychosis and atypical dementia, and 16-year survival. Here, we describe the histopathological and PrP molecular properties in the autopsied brain of this patient. Histopathological examination revealed widespread brain atrophy, focal spongiform degeneration (SD), cortical PrP plaques, and elongated PrP formations in the cerebellum. Overall, these histopathological features resemble those described in a Belgian pedigree with 7-OPRI mutation except for the presence of PrP plaques in our case, which are morphologically different from the multicore plaques described in some OPRI mutations and in Gerstmann–Sträussler–Scheinker (GSS) syndrome. The comparative characterization of the detergent-soluble and detergent-insoluble PrP in our patient and in sporadic Creutzfeldt–Jakob disease (CJD) revealed distinct molecular signatures. Proteinase K digestion of the pathogenic, disease-associated PrP (PrP^D^) revealed PrP^D^ type 1 in the cerebral cortex and mixed PrP^D^ types 1 and 2 in the cerebellum. Altogether, the present study outlines the importance of assessing the phenotypical and PrP biochemical properties of these rare conditions, thereby widening the spectrum of the phenotypic heterogeneity of the 7-OPRI insertion mutations. Further studies are needed to determine whether distinct conformers of PrP^D^ are associated with two major clinico-histopathological phenotypes in prion disease with 7-OPRI.

## Introduction

Human prion diseases can be classified into three groups according to etiology: sporadic, genetic, and acquired by infection (Gambetti et al., 2003; Puoti et al., 2012). While the sporadic form represents the most common human prion disease and accounts for about 85–90% of cases, genetic forms have been described in 10–15% of cases and are typically associated with point mutations in the coding region of the prion protein (PrP) gene (Gambetti et al., 2011; Bonda et al., 2016). Although point mutations are the most common cause of pathogenic mutations, deletion and insertion of extra base pairs are continuously reported (Palmer et al., 1993; Beck et al., 2001; Capellari et al., 2002; Xiao et al., 2013; Areškevičìūtė et al., 2019; Piazza et al., 2020). Furthermore, experiments with primate (Goldfarb et al., 1991) and transgenic mice (Mead et al., 2006) have shown that genetic prion diseases with insertion mutations (gPrD^Ins^) are transmissible.

Insertion mutations involve a region consisting of 27 base pairs (bp) nonapeptide (R1) followed by four 24-bp octapeptide repeats (R2, R2, R3, and R4) with slight variations at the nucleotide level (Goldfarb et al., 1991). These repeats lie in the portion of the PrP gene encompassing codons 51 and 91, a region matching the copper-binding domain of the protein.

As new studies on gPrD^Ins^ are described, the number of octapeptide repeat insertion (OPRI) variants has increased and encompasses cases with 1- to 12-OPRIs. The broad range of possible OPRIs and the methionine (M)/valine (V) polymorphism at codon 129 of the PrP gene confer phenotypic heterogeneity (Parchi et al., 1999; Kong et al., 2004). In addition, the presence of either one or two types (namely, type 1 and type 2) of the disease-associated PrP (PrP^D^) and a low molecular fragment of approximately 7–8 kDa (PrP7-8) of Gerstmann–Sträussler–Scheinker (GSS) disease may contribute to this heterogeneity (Puoti et al., 1999; Cali et al., 2009; Cali et al., 2020).

Correlations between the number of OPRI and phenotypic expression have suggested three groups of OPRI-associated genetic prion diseases: (1) cases harboring 2- or 4-OPRI and Creutzfeldt–Jakob disease (CJD) phenotype with fast disease progression; (2) cases with 5- to 7-OPRI with CJD reminiscent phenotype and slower disease progression; and (3) cases with 8- to 12-OPRI with the GSS phenotype (Kim et al., 2018). However, phenotypic expression in subjects with OPRI does not always fit this general classification (Areškevičìūtė et al., 2019). Moreover, great variability in the clinical and histopathological phenotype has been reported within family members.

The phenotypic expression and the allelic origin of PrP^D^ associated with 7-OPRI have been well characterized (Goldfarb et al., 1991; Tateishi, 1991; Brown et al., 1992; Dermaut et al., 2000; Lewis et al., 2003; Wang et al., 2007; Guo et al., 2008; Mauro et al., 2008; Jansen et al., 2011), and two major clinicopathological disease phenotypes have been described. The first one, reported in a Dutch family, resembles GSS with family members presenting with cognitive and motor impairment at around the fifth decade of life and mean disease duration of ~2.5 years. Major histopathological features include uni- and multicentric PrP plaques with amyloid tinctorial properties and the lack of elongated PrP deposits in the cerebellum, a recurrent feature in gPrD^Ins^ patients. Genetic analysis available in two family members disclosed *cis*-V at PrP-codon 129 (Jansen et al., 2011). The second disease phenotype, described in a Belgian kindred, exhibits cognitive decline at mean age of 29 years and mean disease duration of 13 years. Histopathological examination revealed the lack of PrP plaques and the presence of elongated PrP deposits in the cerebellum; these patients were *cis*-M at PrP-codon 129 (Dermaut et al., 2000). An exception to the aforementioned clinical phenotypes is represented by a Chinese patient with *cis*-M PrP-codon 129 who exhibited memory impairment at age 44 and relatively short disease duration of 4 years. Histopathological examination did not provide information relative to the presence of plaques or elongated PrP deposits (Wang et al., 2007).

In the present study, we describe the histopathological and molecular findings in a young gPrD^Ins^ patient with 7-OPRI mutation and slowly progressive cognitive decline (Mauro et al., 2008). The clinical and most of the histopathological features resemble those described in the Belgium kindred, supporting the diagnosis of genetic CJD. Biochemical characterization of PrP harvested from detergent-soluble and detergent-insoluble fractions unveiled a distinctive signature of the prion protein.

## Results

### Clinical and Genetic Findings

The detailed clinical phenotype and genetic analysis were described in our previous study (Mauro et al., 2008). Briefly, a 19-year-old man presented with psychosis and very slowly progressive atypical dementia, characterized by behavior changes and posterior parietal cognitive signs including visuospatial and constructional deficits, ideomotor apraxia, left–right confusion, and dyscalculia. At the age of 33, the neurological course of the disease changed, becoming rapid and leading the patient to apallic coma in a few months. He died at the age of 34, after 15 years of clinical disease (Supplementary Figure S1).

The diagnosis of genetic prion disease was obtained 8 years from the onset. Sequence analysis on the proband’s DNA demonstrated a 168-bp insertion corresponding to a novel seven extra repeats insertion mutation in the open reading frame of the PrP gene. The 7-OPRI mutation was not present in the patient’s parents.

Although repeats had the same amino acid sequence, they could be discriminated by their DNA sequence. In the mutated allele, repeats were arranged in the following pattern: R1–R2–R2–R3–R2–R2–R3g–R2–R2–R2–R3–R4. Genotype at the polymorphic codon 129 of the PrP gene disclosed methionine homozygosity (Mauro et al., 2008).

### Histopathological Phenotype

**Figure 1:**
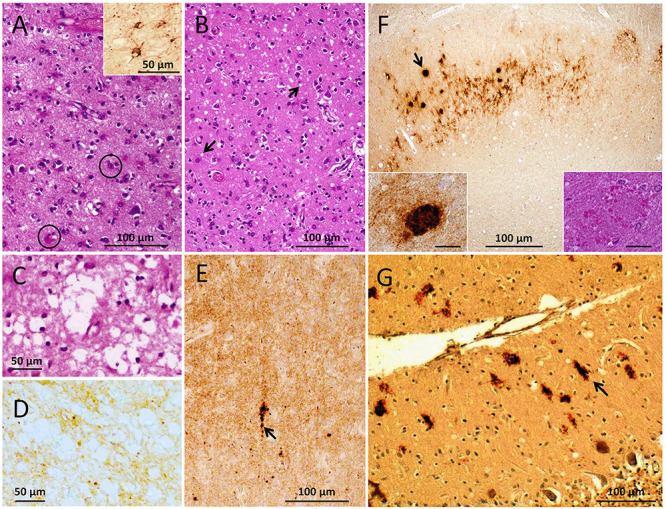
Histopathological phenotype. **(A–C)** Hematoxylin and eosin (HE) staining. **(D–G)** Prion protein (PrP) immunohistochemistry (IHC). **(A)** Severe astrogliosis with reactive astrocytes (circles) affecting the cerebral cortex; inset: glial fibrillary acidic protein (GFAP) immunostaining. **(B)** Spongiform degeneration (SD, arrows) in the parahippocampal gyrus. **(C,D)** Large vacuoles SD **(C)** and diffuse PrP immunostaining **(D)** of the same region (occipital cortex). **(E)** Coarser PrP aggregates (arrow) in a background of diffuse PrP (entorhinal cortex). **(F)** PrP plaque formations (arrow) in a background of diffuse PrP (subiculum); insets: PrP plaque on HE section (right) and after PrP IHC (left). **(G)** Elongated (arrow) and patchy PrP aggregates in the cerebellar molecular layer. Scale bar insets in **(C)**: 20 μm; antibody: 3F4 **(D–G)**.

Overall, a major histopathological feature of our gPrD^Ins^ case was marked atrophy, characterized by neuronal loss and severe astrogliosis throughout the brain (Figure 1A). Spongiform degeneration (SD) was focal with small vacuoles affecting the cerebral neocortex and the parahippocampal gyrus (Figure 1B). Spongiosis was occasionally pronounced in layer II of the cerebral cortex; scattered vacuoles were noted in the cerebellar molecular layer. Unlike other cortical regions, SD in the occipital cortex, and to a lesser extent the parietal cortex, showed few areas with larger and confluent vacuoles associated with more severe gliosis and neuronal loss (Figure 1C). Eosinophilic plaque formations without a dense core were conspicuous in the subiculum and rare in the temporal neocortex (inset of Figure 1F). The cerebellum was characterized by moderate atrophy with loss of Purkinje and granule cells.

Immunohistochemical examination for glial fibrillary acidic protein (GFAP) revealed an intense astrocytic reaction (inset of Figure 1A). Immunostaining for PrP showed scattered small aggregates—sometimes with the appearance of plaque-like formations—in a background of diffuse PrP in the cerebral cortex, including the dentate gyrus of the hippocampus, basal ganglia, and brainstem (Figures 1D,E). The CA4 region of the hippocampus and the entorhinal cortex showed diffuse and perineuronal PrP deposits. In cortical region with large vacuoles PrP immunostaining was of the diffuse type with rare coarser granules (Figure 1D). The eosinophilic plaque formations detected on hematoxylin–eosin sections reacted with an anti-PrP antibody (Figure 1F), while elongated and truncated PrP formations with orientation perpendicular to the leptomeningeal surface populated the molecular layer of the cerebellum (Figure 1G).

### Western Blot Profiles and PK-Titration Assay of PrP

**Figure 2:**
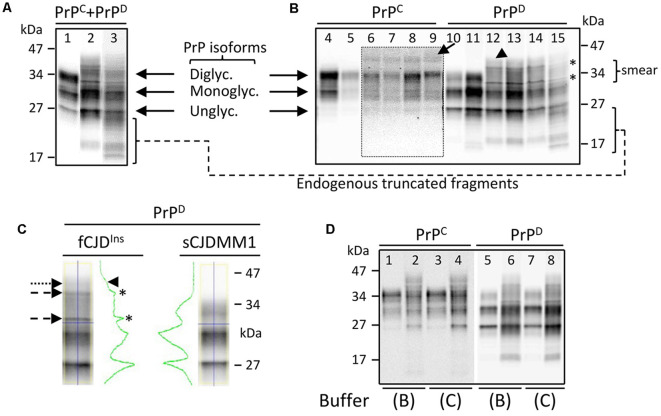
Western blot (WB) profiles of detergent-soluble and detergent-insoluble prion protein (PrP). PrP bands were resolved in a 15% Tris–HCl, 8.7-cm-long gel and visualized with the near-infrared LI-COR system.** (A)** Brain homogenate (S1) containing both PrP^C^ and PrP^D^ species (PrP^C^ + PrP^D^) showing three major fragments corresponding to di-, mono- and unglycosylated PrP isoforms in sCJDMM1 (lane 1). A PrP smear in the ~30–45 kDa area of the gel is observed in addition to mono- and unglycosylated PrP isoforms in fCJD^Ins^ (lanes 2 and 3). Bands in the ~17–25 kDa regions represent endogenously truncated PrP fragments; frontal cortex (cx; lanes 1 and 2) and cerebellum (lane 3). **(B)** PrP^C^ shows one band in the ~42–44 kDa region of the gel (arrow) in addition to di-, mono- and unglycosylated PrP^C^ in gPrD^Ins^ (lanes 6–9). WB profiles of PrP^C^ from sCJDMM1 (lane 4) and sCJDVV2 (lane 5) show the typical three PrP^C^ isoforms. The inset represents a longer exposure of area highlighted by the dotted rectangle. The detergent-insoluble PrP^D^ shows a smear and two sharp bands (asterisks) between ~32 and ~40 kDa, and a higher faint band of ~42–44 kDa (arrowhead) in gPrD^Ins^ (lanes 12–15). PrP^D^ in sCJDMM1 (lane 10) and sCJDVV2 (lane 11) is characterized by the typical three PrP^D^ isoforms; frontal cx (lanes 4–6, 10–12); parietal cx (lanes 7 and 13); occipital cx (lanes 8 and 14); cerebellum (lanes 9 and 15). **(C)** PrP^D^ harvested from gPrD^Ins^ and sCJDMM1 shows distinct WB profiles as highlighted by the green line: the two small peaks (asterisks) and the slope (arrowhead) in PrP^D^-gPrD^Ins^ are generated by the two sharp fragments (dashed arrows) and a smear (dotted arrow), respectively. **(D)** WB profiles of PrP^C^ and PrP^D^ obtained from gPrD^Ins^ (lanes 2, 4, 6, 8) and sCJDMM1 (lanes 1, 3, 5, 7); samples were prepared using two buffers (buffers B and C; see Methods and Supplementary Figure S2) with high percentage of a detergent. The same loading was used in each WB in panels **(A–D)**; antibody: 3F4.

Brain homogenates (S1) harvested from gPrD^Ins^ showed the typical mono- and unglycosylated PrP isoforms migrating to ~30 and ~27 kDa, respectively, similar to PrP in sCJDMM1—a sCJD subtype with PrP-129MM genotype and PrP^D^ type 1 (Figure 2A). Unlike sCJD, however, PrP from gPrD^Ins^ was characterized by the lack of a well-defined diglycosylated isoform. Furthermore, a smear of faint bands in the ~32–40 kDa range migrated above the monoglycosylated PrP isoform in gPrD^Ins^ but not in sCJD controls (Figure 2A). PrP fragments ranging between ~17 and ~25 kDa were abundant in gPrD^Ins^ (Figure 2A). These fragments were not detected when probing with an antibody (Ab) to an epitope located to the PrP N-terminal region (residues 36–43), indicating that these endogenous fragments are N-terminally truncated (data not shown). In order to have a more detailed picture of the PrP Western blot (WB) profile, S1 preparations were subjected to high-speed centrifugation to separate the detergent-soluble (S2, containing wild-type and mutated PrP^C^) from detergent-insoluble (P2, containing wild-type and mutated PrP^D^) fraction (Figures 2B–D). In gPrD^Ins^ only, PrP^C^ showed a faint band of ~42–44 kDa in addition to di- (~34 kDa) and mono-glycosylated (~30 kDa) PrP isoforms, while the unglycosylated (~27 kDa) PrP isoform was better visualized at longer signal captures (Figure 2B).

The pathogenic PrP^D^ harvested from the detergent-insoluble fraction showed two sharp fragments in the ~32–40 kDa region lying in a background of PrP smear in gPrD^Ins^ but not in sCJD (Figures 2B,C). The ~42–44 kDa high molecular fragment populated also PrP^D^. Notably, the composition of the buffer and the amount of detergent did not change the molecular profiles of PrP^C^ and PrP^D^ species (Figure 2D).

**Figure 3:**
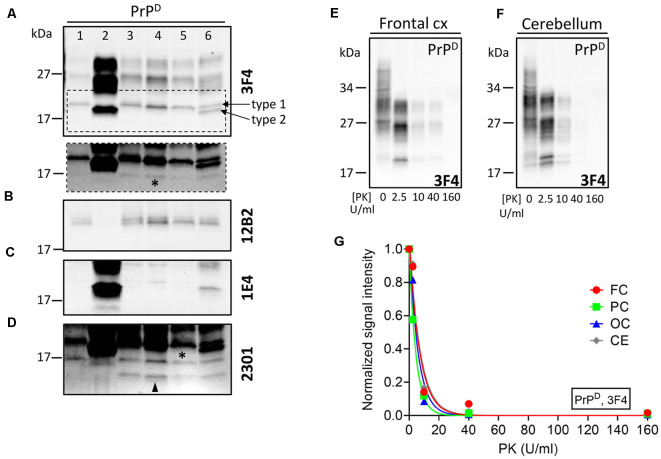
WB profiles of resPrP^D^ and proteinase K (PK)-titration assay of PrP^D^. PrP^D^ from gPrD^Ins^ (lanes 3–6), sCJDMM1 (lane 1), and sCJDVV2 (lane 2) was digested with PK at concentration of 10 U/ml PK and probed with antibodies **(A,E–G)** 3F4, **(B)** 12B2, **(C)** 1E4, and **(D)** 2301. **(A)** The unglycosylated resPrP^D^ isoform from the neocortex of gPrD^Ins^ (lanes 3–5) and resPrP^D^ T1 from sCJDMM1 (lane 1) comigrates to ~20 kDa. In the cerebellum of gPrD^Ins^ (lane 6), the ~20-kDa fragment coexists with a ~19-kDa band matching in mobility resPrP^D^ T2 of sCJDVV2 (lane 2). Dashed rectangle: longer exposure of the area highlighted in the main figure showing low amounts of a ~17-kDa fragment (asterisk) in the cerebral cortex and cerebellum; frontal (lane 3), parietal (lane 4), and occipital (lane 5) cortices. **(B)** The T1-selective 12B2 Ab immunoreacts with T1 (lanes 1, 3–6) but not with T2 (lanes 2 and 6). **(C)** The Ab 1E4 efficiently binds to T2 (lanes 2 and 6) and only weakly with T1 (lane 4). **(D)** The 2301 Ab immunoreacts with resPrP^D^ of ~17 kDa (asterisk) and resPrP^D^ CTF ~12/13 kDa (arrowhead). **(E,F)** Representative WB showing PrP^D^-gPrD^Ins^ subjected to PK digestion at concentration of 0, 2.5, 10, 40, and 160 U/ml. **(G)** PK_1/2_ (corresponding to the PK concentration required to digest half of PrP^D^) was similar in the brain regions of gPrD^Ins^ [4.3 ± 0.4 U/ml expressed as mean ± SEM of frontal cortex (FC), parietal cortex (PC), occipital cortex (OC) and cerebellum (CE)].

Proteinase K (PK) digestion of PrP^D^ generated truncated PK-resistant PrP^D^ (resPrP^D^) corresponding to di- (~30–31 kDa), mono- (~27 kDa), and unglycosylated resPrP^D^ isoforms. The unglycosylated resPrP^D^ band, a surrogate marker for prion strains, migrated as a single band of ~20 kDa in the cerebral cortex of gPrD^Ins^, matching the gel mobility of resPrP^D^ type 1 (T1; Figure 3A; Cali et al., 2006, 2009). On the contrary, two co-existing resPrP^D^ bands of ~20 and ~19 kDa were found in the cerebellum; the lower band comigrated with resPrP^D^ type 2 of sCJDVV2–a sCJD subtype with PrP-129VV genotype and PrP^D^ type 2 (Figure 3A). The T1-selective 12B2 Ab confirmed the presence of resPrP^D^ T1 in gPrD^Ins^ and sCJDMM1 but not in sCJDVV2 (Figure 3B), whereas the 1E4 Ab, which preferentially binds to type 2 (T2), detected resPrP^D^ T2 in the cerebellum but not in the cerebral cortex of gPrD^Ins^ (Figure 3C). Finally, the 2301 Ab immunoreacting with the C-terminal portion of PrP (residues 220–231) detected similar amounts of ~13 kDa, and possibly ~12 kDa, C-terminal fragments (referred as to PrP-CTF 12/13) in gPrD^Ins^ (Figure 3D; Zou et al., 2003).

PK-titration assay of PrP^D^ harvested from the cerebral cortex and cerebellum showed similar profiles. PK_1/2_—the amount of PK required to digest 50% of PrP^D^—ranged between 3 U/ml (parietal cortex) and 5 U/ml (frontal cortex and cerebellum; Figures 3E–G).

## Discussion

Here, we carried out a description of the histopathological and biochemical features of a novel *de novo* 7-OPRI mutation in a patient presenting with early onset behavioral changes and long survival (Mauro et al., 2008). Overall, the age at onset (19 years) and disease duration (16 years) are in line with the clinical data reported in families with 7-OPRI mutation coupled with methionine at PrP-codon 129 of the mutated allele (*cis*-M; Dermaut et al., 2000; Lewis et al., 2003). However, we do not know whether the co-occurrence of PrP^D^ types and/or the novel sequence of the extra repeats in our case contributed to further anticipate the age at onset of the youngest patient with 7-OPRI mutation. According to Lewis et al. (2003), the presence of valine in the normal allele (*trans*-V) coupled with PrP^D^ T2 does not affect age at onset and disease duration clinical features. A marked exception to the abovementioned clinical features is represented by a *cis*-M member of a Chinese family who presented with memory deficit at older age (46 years) and had relatively shorter disease course (~4 years; Wang et al., 2007). Similar to other patients carrying the 7-OPRI mutation, cognitive decline and apraxia were among the clinical features in our case. However, personality changes with autistic-like behavior at onset, marked parietal atrophy, and absence of cerebellar ataxia represent novelties.

The evaluation of the histopathological phenotype revealed some characteristics matching those described by Dermaut et al. (2000), including: (i) a generalized brain atrophy; (ii) mild spongiosis with preferential distribution in the cortical layer II; (iii) absence of SD in the hippocampus (CA1–CA4); (iv) loss of Purkinje cells; and (v) presence of elongated PrP deposits in the cerebellar molecular layer. However, a distinguishing phenotypical feature is represented by the core-free PrP cortical plaques in our case; these plaques are morphologically different from the unicentric and multicore (Gelpi et al., 2005; Jansen et al., 2011; Xiao et al., 2013), kuru (Tateishi, 1991; Xiao et al., 2013), and florid plaques (Pietrini et al., 2003) reported in cases with various OPRI mutations. Histopathological characteristics as those described by Lewis et al. (2003) in a patient with 7-OPRI mutation only partially resemble those of our and Dermaut’s cases. Genetic differences in the OPRI and the presence of valine in the normal allele may account for the lack of elongated cerebellar PrP deposits (also referred to as “stripes”) in the study of Lewis et al. (2003).

The presence of elongated cerebellar PrP deposits in our and other cases, a pathognomonic feature of the OPRI mutation, has been described in cases with different PrP-codon 129 genotypes and PrP^D^ types (Capellari et al., 1997; Dermaut et al., 2000; Mead et al., 2007; Jansen et al., 2009; Xiao et al., 2013; Areškevičìūtė et al., 2019). Notably, elongated PrP deposits have been reported in cases with 5- and 6-OPRI mutations lacking detectable resPrP^D^, suggesting that the PK-sensitive portion of PrP^D^ is sufficient to initiate and sustain neurodegeneration in these patients (Mead et al., 2007; Xiao et al., 2013).

Histopathologically, large vacuoles and coarse and/or perivacuolar PrP deposition are cardinal features of sCJDMM2 and sCJDMV2C subtypes (Parchi and Saverioni, 2012; Baiardi et al., 2019) as well as sCJD cases with mixed PrP^D^ types (e.g., sCJDMM1-2 and sCJDMV1-2C; Puoti et al., 1999; Cali et al., 2009; Parchi et al., 2009). Unlike the above sCJD subtypes, PrP immunostaining of cortical regions with large vacuoles was of the diffuse type in our case. The lack of coarse/perivacuolar PrP deposits in regions with large vacuoles may not be uncommon in some prion diseases including gPrD^Ins^ (Cali et al., 2018).

The complexity of the electrophoretic profile of PrP from various gPrD^Ins^ is undoubtedly generated by the extra octapeptide repeats (Capellari et al., 1997; Lewis et al., 2003; Pietrini et al., 2003; Gelpi et al., 2005; Mead et al., 2007; Xiao et al., 2013). As expected, the WB profiles of PrP^C^ and PrP^D^ differed from those of sCJD. These differences included the presence of a high molecular weight fragment in the mutated PrP, which likely represents the mutated diglycosylated PrP isoform; mutated mono- and unglycosylated PrP isoforms should be interspersed within the wild-type PrP. To determine the glycosylation nature of two sharp PrP^D^ bands in the ~32–40 kDa region and the lack of a well-defined diglycosylated PrP^D^ isoform, additional investigations would be required. Importantly, WB profiles of the mutated PrP^C^, and of the resulting PrP^D^, were reproducible under different experimental conditions. Furthermore, the apparently higher levels of endogenously truncated PrP^D^ fragments (spanning ~17–25 kDa) in our case compared to sCJD is in agreement with a previous study (Gelpi et al., 2005) and highlights the propensity of gPrD^Ins^ cases to generate truncated PrP^D^ fragments.

Proteinase K digestion of PrP^D^ generated similar ratios of di-, -mono-, and unglycosylated resPrP^D^ in gPrD^Ins^ and sCJD. In patients with different OPRI mutations, coexisting resPrP^D^ types T1 and T2 (T1–2) have been reported in the same brain region or separately in different anatomical locations (Pietrini et al., 2003; Jansen et al., 2009). In our case, both resPrP^D^ types were found in the cerebellum, whereas T1, but not T2, was present in the cerebral cortex. The reason for the lack of T2 detection in the cerebral cortex could be due to the limited number of cortical regions assessed and small amount of tissue used (Cali et al., 2009, 2020). Furthermore, the presence of coarse SD in the cerebral cortex, although focal, suggests that a minor component of T2 is present.

As a measure of the conformation of PrP^D^, we recently determined the resistance to proteolysis by digestion with PK of PrP^D^ T1-2 in patients with sCJDVV1-2 (Cali et al., 2020). From this study, we found that the PK_1/2_ index of T1–2 was significantly greater than PrP^D^ T1. Here, the PK_1/2_ of PrP^D^ T1–2 and T1 were virtually identical, suggesting conformational differences of T1–2 between gPrD^Ins^ and sCJD. However, the small number of brain regions with T1–2 in our case and some modifications in the protocol may limit this interpretation.

Finally, we did not detect the ~7–8 kDa PrP^D^ fragment described in GSS and in a gPrD^Ins^ with 7-OPRI mutation and GSS-like histopathological phenotype (Gelpi et al., 2005; Jansen et al., 2011). The lowest molecular weight PrP fragment in gPrD^Ins^ with 6-OPRI mutation described by Gelpi et al. may correspond to the ~7–8 kDa fragment. As demonstrated by these two reports as well as in a GSS study, the ~7–8 kDa PrP band immunoreacts well with an antibody to the central portion of PrP (e.g., 3F4; Parchi et al., 1998; Cracco et al., 2019). Although it is not known with certainty whether the presence of the 7–8 kDa PrP band is invariably associated with the GSS phenotype in gPrD^Ins^ (Xiao et al., 2013), the lack of this fragment and the different morphologies of the plaques point towards a novel phenotype of our gPrD^Ins^ case.

Overall, the present case study broadens the spectrum of phenotypic expression of the inherited prion disease linked to 7-extra octapeptide repeats insertion mutation. The presence of amorphous PrP plaques in the cerebral cortex adds to the phenotypic variability of cases with the same mutation. Furthermore, this study highlights the importance of determining the biochemical properties of the soluble and insoluble PrP species as the molecular determinant of phenotypic expression in prion diseases.

## Data Availability Statement

The raw data supporting the conclusions of this article will be made available by the authors, without undue reservation.

## Ethics Statement

Written informed consent was obtained from the individuals for the publication of this case report, including any potentially identifiable images or data contained in this article.

## Author Contributions

IC and GP conceived and designed the experiments. IC, LC, DS, BSA, and GP performed the experiments. IC, LC, DS, RO, CC, and GP analyzed the data. IC and GP wrote the manuscript. All authors reviewed the manuscript.

## Conflict of Interest

The authors declare that the research was conducted in the absence of any commercial or financial relationships that could be construed as a potential conflict of interest.
