# Supplementary material for: Case Report: Histopathology and Prion Protein Molecular Properties in Inherited Prion Disease With a De Novo Seven-Octapeptide Repeat Insertion
Source: Front Cell Neurosci. 2020 Jul 8;14:150. doi: 10.3389/fncel.2020.00150 (PMC7362343; doi:10.3389/fncel.2020.00150)
Supplement: Supplementary file 1 [file Table_1.docx]

**MATERIALS AND METHODS**

**Reagents and antibodies**

Phenylmethanesulfonyl fluoride (PMSF), 10X Dulbecco’s Phosphate Buffered Saline (DPBS), proteinase K (PK), Sarkosyl NL and glial fibrillary acidic protein (GFAP) antibody (Ab) were from Sigma-Aldrich (St. Louis, MO, USA). Tween 20, 10X Tris-buffered saline (TBS), 2X Laemmli Sample Buffer and 15% Tris-HCl polyacrylamide Criterion precast gels were from Bio-Rad Laboratories (Hercules, CA, USA). Odyssey blocking buffer and Infrared Dye (IRDye) 800CW goat anti-mouse IgG and IRDye 680CW goat anti-rabbit IgG (1 mg/ml) were from LI-COR Biosciences (Lincoln, NE, USA). Polyvinylidene fluoride (PVDF) membrane (Immobilon-FL) was from EMD Millipore (Billerica, MA, USA). The following monoclonal Abs to PrP were used: 8B4 to PrP epitope 36-43 (Li *et al.*, 2000), 12B2 to PrP epitope 89-93 (Langeveld *et al.*, 2006), 1E4 to PrP epitope 97-108(Cali *et al.*, 2020) (Cell Sciences, Canton, MA, USA), and 3F4 to PrP epitope 106-110 (Kascsak *et al.*, 1987; Zou *et al.*, 2010). The anti-PrP polyclonal Ab included the rabbit antiserum 2301 to PrP residues 220-231 (Chen *et al.*, 1995).

**Brain tissue**

Brain autopsy of the propositus was performed at the Prion Disease Diagnosis and Surveillance Center (PDDSC) in the Department of Advanced Medical and Surgical Sciences, University of Campania “Luigi Vanvitelli” (Caserta, Italy) (Mauro *et al.*, 2008), while the molecular and pathological studies were carried out at the National Prion Disease Pathology Surveillance Center (NPDPSC) at Case Western Reserve University (Cleveland, USA). Control cases included one sCJDMM1 and one sCJDVV2 – two common subtypes of sCJD (Parchi *et al.*, 1996, 1999) – obtained from the NPDPSC’s Brain Bank. At autopsy, half brain was stored at -80 °C whereas the other half was fixed either in Carnoy's fixing solution (gPrD^Ins^) or in formalin (sCJD controls) as previously described (Giaccone *et al.*, 2000; Cali *et al.*, 2020). Frozen brain tissue examined included the frontal, parietal and occipital cortices and cerebellum in the gPrD^Ins^, and the frontal cortex in sCJD controls.

**Phenotype characterization: histology and PrP immunohistochemistry**

Formalin-fixed brain tissue was treated as previously described (Cali *et al.*, 2020). Sections from each brain region were deparaffinized, rehydrated, and immersed in 1X Tris buffered saline containing Tween 20 (TBS-T). Endogenous peroxidase was blocked by the Envision Flex Peroxidase Blocking Reagent (Dako North America Inc., Carpinteria, USA) for 10 minutes (min) and washed. Sections were immersed in 1.5 mmol/L hydrochloric acid, microwaved for 15 min and probed with the Abs 3F4 (1:1,000) or GFAP (1:12,000) for 1 hour (h). Following this procedure, sections were washed again, then incubated with Envision Flex/HRP polymer for 30 min (Dako). Envision Flex DAB (Dako) was used to visualize the immunostaining.

**Preparation of the detergent-soluble and detergent-insoluble fractions**

Frozen brain tissue was homogenized in 1X DPBS, pH 7.4 to make a 20% (weight/volume) brain homogenate (BH) prior to dilution with an equal volume of 2X lysis buffer 100 (1X LB100: 100 mM NaCl, 0.5% Nonidet P-40, 0.5% sodium deoxycholate, 10 mM EDTA, 100 mM Tris-HCl, pH 8.0; Buffer A) (Cali *et al.*, 2015). The resulting 10% BH was centrifuged at low speed (1,000 x g) for 5 min at 4 ⁰C and the supernatant (S1) was collected. The S1 was subjected to high speed centrifugation (100,000 x g) for 1 h at 4 ⁰C to separate the detergent-soluble (S2) from the detergent-insoluble (P2) fraction. The pellet (P2) was re-suspended by sonication in 1X LB100 pH 8.0. Aliquots of S2, which contain the physiological or cellular PrP (PrP^C^) were mixed with 5-fold pre-chilled methanol for 2 h and centrifuged at 17,200 x g for 30 min at 4 °C. The resulting pellets were re-suspended in 1X LB100. In a control experiment (Figure 2 D and Suppl. Figure S2), the 20% BH was incubated with an equal volume of 16% sarkosyl NL-2X LB100 pH 8.0 (Buffer B) or in 16% sarkosyl NL-1X DPBS pH 7.4 (8% sarkosyl NL final concentration) (Buffer C) at 4 ⁰C prior to low speed centrifugation (1,000 x g; S1). The S1 was then subjected to high speed (100,000 x g) centrifugation to separate the S2 and P2 fractions.

**PK digestion, PK-titration assay, and Western blot (WB) analysis**

Aliquots of S1, S2, and P2 were left either untreated or treated with 10 units/ml (U/ml) PK [48 U/mg specific activity, 1 U/ml equal to 20.8 µg/ml PK] at 37 ⁰C for 1 h with agitation. For the PK-titration assay, PK was used at concentrations of 0, 2.5, 10, 40 and 160 U/ml. The enzymatic reaction was stopped by addition of 3mM PMSF. Each sample was mixed with an equal volume of 2X Laemmli sample buffer and denatured for 10 min at 100°C. Proteins were separated using 15% Tris-HCl polyacrylamide Criterion precast gels and transferred into Immobilon-FL PVDF membranes for 2 h. Membranes were blocked with the Odyssey Blocking Buffer for 1 h prior incubation with the primary antibody for 2 h. Antibodies 8B4, 3F4, 12B2, 1E4 and 2301 were diluted 1:5,000, 1:20,000, 1:12,000, 1:1,000 and 1:5:000, respectively in Odyssey Blocking Buffer mixed with Tween 20 (0.1% final concertation). Membranes were washed with 1X DPBS containing 0.1% Tween 20 and incubated for 1h with IRDye 800CW goat anti-mouse IgG (1:15,000) or IRDye 680CW goat anti-rabbit IgG (1:15,000) secondary Abs that was diluted in Odyssey Blocking Buffer containing 0.1% Tween 20 and 0.01% SDS. After washing, membranes were developed by the Odyssey near-infrared imaging system (LI-COR Biosciences). Densitometric analysis was performed using the Odyssey application software V3.0 (LI-COR Biosciences). In the PK titration assay, the five PK points were best fitted by a one-phase decay equation (Cali *et al.*, 2020), and the PK_1/2_, defined as the amount of PK required to hydrolyze 50% of PrP^D^, was calculated with the software GraphPad Prism 8.1.1. For the majority of the experiments, WB were performed in triplicate.

**Genetics**

Genetic analysis of the PrP gene was performed as previously described (Mauro *et al.*, 2008).

**BIBLIOGRAPHY**

Cali I, Miller CJ, Parisi JE, Geschwind MD, Gambetti P, Schonberger LB. Distinct pathological phenotypes of Creutzfeldt-Jakob disease in recipients of prion-contaminated growth hormone. Acta Neuropathol Commun 2015; 3: 37.

Cali I, Puoti G, Smucny J, Curtiss PM, Cracco L, Kitamoto T, et al. Co-existence of PrPD types 1 and 2 in sporadic Creutzfeldt-Jakob disease of the VV subgroup: phenotypic and prion protein characteristics. Sci Rep 2020; 10: 1503.

Chen SG, Teplow DB, Parchi P, Teller JK, Gambetti P, Autilio-Gambetti L. Truncated forms of the human prion protein in normal brain and in prion diseases. J Biol Chem 1995; 270: 19173–19180.

Giaccone G, Canciani B, Puoti G, Rossi G, Goffredo D, Iussich S, et al. Creutzfeldt-Jakob disease: Carnoy’s fixative improves the immunohistochemistry of the proteinase K-resistant prion protein. Brain Pathol 2000; 10: 31–37.

Kascsak RJ, Rubenstein R, Merz PA, Tonna-DeMasi M, Fersko R, Carp RI, et al. Mouse polyclonal and monoclonal antibody to scrapie-associated fibril proteins. J Virol 1987; 61: 3688–3693.

Langeveld JPM, Jacobs JG, Erkens JHF, Bossers A, van Zijderveld FG, van Keulen LJM. Rapid and discriminatory diagnosis of scrapie and BSE in retro-pharyngeal lymph nodes of sheep. BMC Vet Res 2006; 2: 19.

Li R, Liu T, Wong BS, Pan T, Morillas M, Swietnicki W, et al. Identification of an epitope in the C terminus of normal prion protein whose expression is modulated by binding events in the N terminus. J Mol Biol 2000; 301: 567–573.

Mauro C, Giaccone G, Piscosquito G, Lavorgna A, Nigro M, Di Fede G, et al. A novel insertional mutation in the prion protein gene: clinical and bio-molecular findings. J Neurol Neurosurg Psychiatry 2008; 79: 1395–1398.

Parchi P, Castellani R, Capellari S, Ghetti B, Young K, Chen SG, et al. Molecular basis of phenotypic variability in sporadic Creutzfeldt-Jakob disease. Ann Neurol 1996; 39: 767–778.

Parchi P, Giese A, Capellari S, Brown P, Schulz-Schaeffer W, Windl O, et al. Classification of sporadic Creutzfeldt-Jakob disease based on molecular and phenotypic analysis of 300 subjects. Ann Neurol 1999; 46: 224–233.

Zou W-Q, Langeveld J, Xiao X, Chen S, McGeer PL, Yuan J, et al. PrP conformational transitions alter species preference of a PrP-specific antibody. J Biol Chem 2010; 285: 13874–13884.

Supplementary Figure S1. **Timeline of clinical evolution in our patient**. The timeline includes clinical findings at disease onset, clinical diagnosis at various ages, follow-up period, and age at death. Numbers within circles identify the age of the patient; y: years.

Supplementary Figure S2. **Flow diagram of PrP centrifugation**. The following preparations were used: S1, obtained at low speed centrifugation (1,000 x g); S2 or detergent-soluble fraction obtained following high speed centrifugation (100,000 x g) and containing the cellular PrP (PrP^C^); P2 or the detergent-insoluble or pellet fraction (100,000 x g) contains the pathological or disease-related PrP (PrP^D^). Buffers B and C have high content of the detergent Sarkosyl. Mut.: mutated; wt: wild-type.
