# Supplementary figures and images for: Case Report: Histopathology and Prion Protein Molecular Properties in Inherited Prion Disease With a De Novo Seven-Octapeptide Repeat Insertion
Source: Front Cell Neurosci. 2020 Jul 8;14:150. doi: 10.3389/fncel.2020.00150 (PMC7362343; doi:10.3389/fncel.2020.00150)

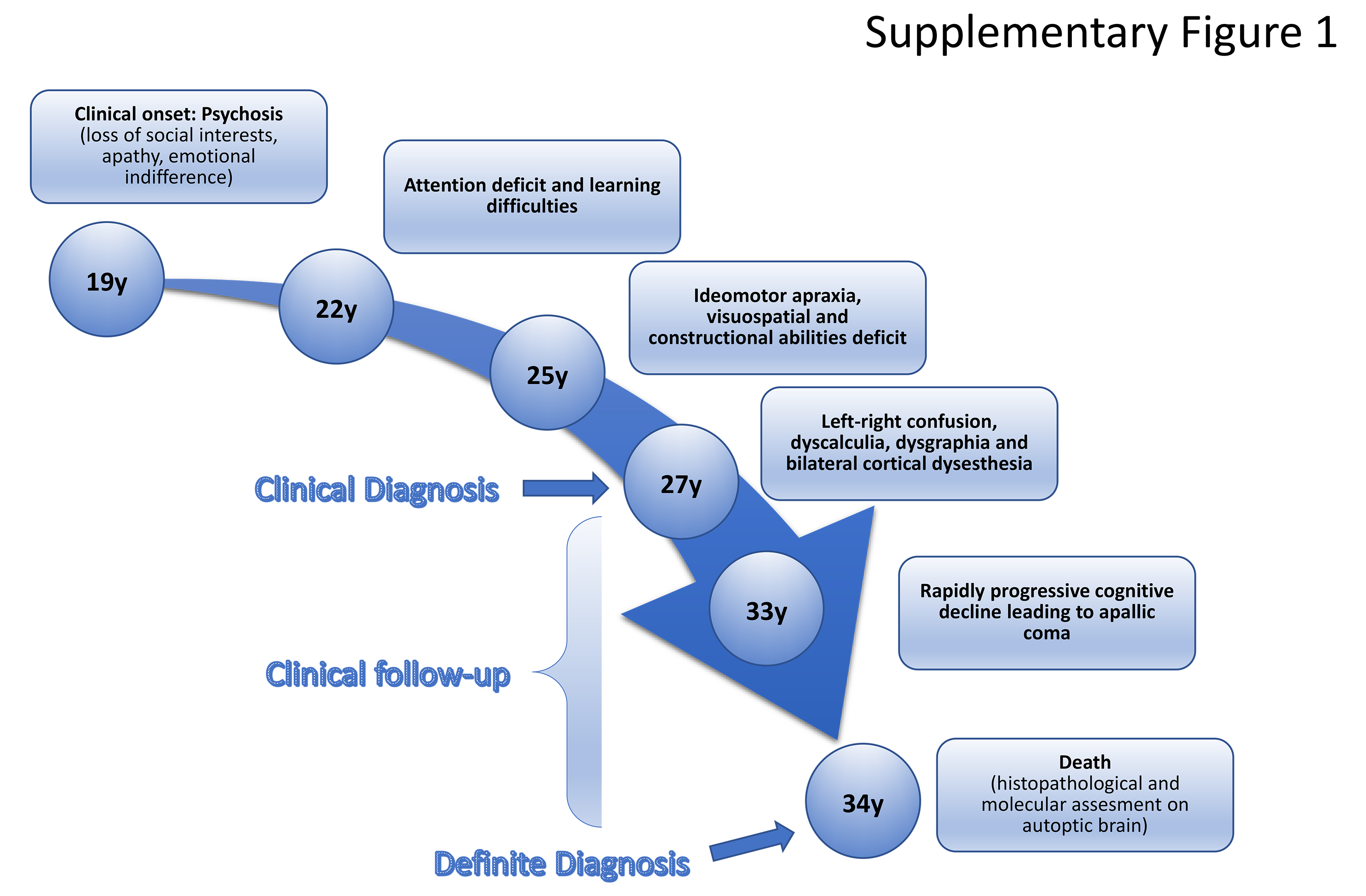

Supplement: Supplementary file 2 [file Image_1.JPEG]

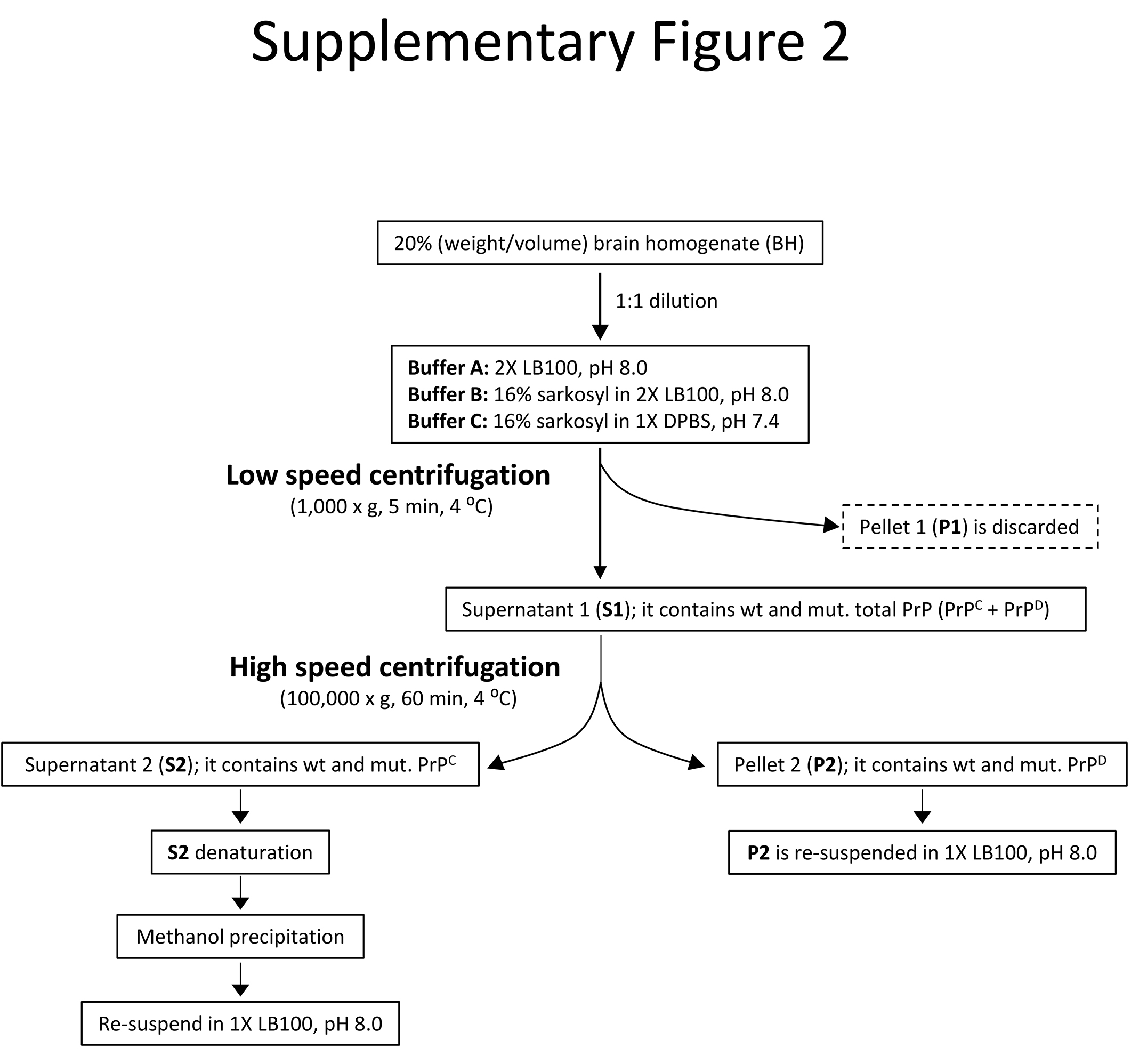

Supplement: Supplementary file 3 [file Image_2.JPEG]
